# AMPA Receptors Commandeer an Ancient Cargo Exporter for Use as an Auxiliary Subunit for Signaling

**DOI:** 10.1371/journal.pone.0030681

**Published:** 2012-01-24

**Authors:** Nadine Harmel, Barbara Cokic, Gerd Zolles, Henrike Berkefeld, Veronika Mauric, Bernd Fakler, Valentin Stein, Nikolaj Klöcker

**Affiliations:** 1 Institute of Neuro- and Sensory Physiology, Medical Faculty, University of Düsseldorf, Düsseldorf, Germany; 2 Department of Synaptic Receptor Trafficking, Max-Planck-Institute of Neurobiology, Martinsried, Germany; 3 Institute of Physiology, University of Freiburg, Freiburg, Germany; 4 Center for Biological Signaling Studies (BIOSS), Freiburg, Germany; 5 Department of Physiology, University of Bonn, Bonn, Germany; Virginia Commonwealth University, United States of America

## Abstract

Fast excitatory neurotransmission in the mammalian central nervous system is mainly mediated by ionotropic glutamate receptors of the AMPA subtype (AMPARs). AMPARs are protein complexes of the pore-lining α-subunits GluA1-4 and auxiliary β-subunits modulating their trafficking and gating. By a proteomic approach, two homologues of the cargo exporter cornichon, CNIH-2 and CNIH-3, have recently been identified as constituents of native AMPARs in mammalian brain. In heterologous reconstitution experiments, CNIH-2 promotes surface expression of GluAs and modulates their biophysical properties. However, its relevance in native AMPAR physiology remains controversial. Here, we have studied the role of CNIH-2 in GluA processing both in heterologous cells and primary rat neurons. Our data demonstrate that CNIH-2 serves an evolutionarily conserved role as a cargo exporter from the endoplasmic reticulum (ER). CNIH-2 cycles continuously between ER and Golgi complex to pick up cargo protein in the ER and then to mediate its preferential export in a coat protein complex (COP) II dependent manner. Interaction with GluA subunits breaks with this ancestral role of CNIH-2 confined to the early secretory pathway. While still taking advantage of being exported preferentially from the ER, GluAs recruit CNIH-2 to the cell surface. Thus, mammalian AMPARs commandeer CNIH-2 for use as a bona fide auxiliary subunit that is able to modify receptor signaling.

## Introduction

In the mammalian CNS, fast excitatory neurotransmission is mainly mediated by ionotropic glutamate receptors of the AMPA subtype (AMPARs). They conduct cation currents under conditions of basal neuronal activity and determine largely the strength of excitatory glutamatergic synapses. Changes in synaptic AMPAR density and their gating properties are centrally involved in forms of synaptic plasticity [1]–[3].

AMPARs form as heterotetramers of the four pore-lining α-subunits GluA1–4, which are differentially expressed in the mammalian brain. Alternative splicing and RNA editing further enhance their diversity with respect to trafficking and biophysical properties [4]–[12]. The GluA subunits co-assemble with transmembrane AMPAR regulatory proteins (TARPs) that modulate both the subcellular distribution and the biophysical properties of native AMPAR complexes [13]–[16]. Stargazin (γ-2), the prototypical TARP, enhances surface expression of AMPARs, their synaptic targeting and recycling by interaction with the postsynaptic scaffolding protein PSD-95 [17]–[19]. Moreover, TARPs increase charge transfer through individual AMPARs as they slow channel deactivation and desensitization and reduce current rectification by polyamines [20], [21].

Recent proteomic approaches identified further auxiliary subunits: the cornichon homologues CNIH-2 and CNIH-3, as well as the Cystine-Knot AMPAR Modulating Protein CKAMP44 [22], [23]. CKAMP44 displays a very distinct pattern of expression in the dentate gyrus of the hippocampus, while the two cornichon isoforms are expressed throughout the brain and are associated with the majority of AMPARs. Both auxiliary subunits affect the gating properties of the GluA subunits: CKAMP44 delays recovery of the receptors from desensitization, CNIH-2/3 slow deactivation and desensitization kinetics. However, whereas CKAMP44 has been implicated in hippocampal short-term plasticity, a physiological role of CNIH-2/3 remains controversial.

The product of the *cornichon* gene was originally identified as being required for correct growth factor signaling during oogenesis [24]. Follow-up studies in drosophila, chicken and transfected culture cells identified cornichon and its orthologues as endoplasmic reticulum (ER) cargo exporters for members of the transforming growth factor α (TGFα) family [25]–[27]. In agreement with these studies, Shi and co-workers have recently suggested that CNIH-2 may exert a chaperone-like function facilitating the surface transport of AMPARs; the physiological relevance of the CNIH-2-mediated effects on receptor gating was questioned, as the authors failed to detect CNIH-2 on the cell surface of neurons [28]. In contrast, Kato et al. using an elegant biophysical approach together with immunocytochemistry demonstrated that CNIH-2 co-assembles into postsynaptic AMPAR complexes and modulates channel gating, pharmacology and association of GluA and TARP subunits [16], [29].

In the present study, we have picked up this debate and investigated the role of CNIH-2 in AMPAR processing in both heterologous and primary cells. Employing cell biological and electrophysiological techniques, we demonstrate that interaction with AMPARs has converted the cargo exporter CNIH-2 usually cycling in the early secretory pathway into a surface membrane protein that is able to modify native AMPAR signaling.

## Results

### CNIH-2 increases functional surface expression of GluAs

**Figure 1:**
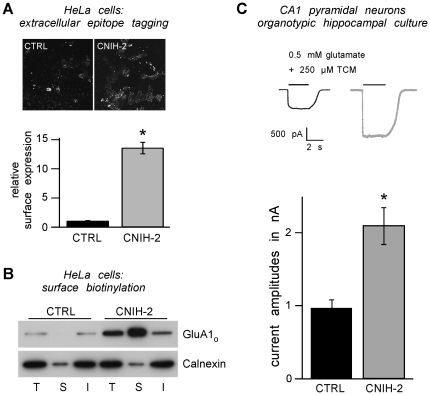
CNIH-2 increases surface expression of AMPARs. **A** Quantification of GluA1_o_ surface expression levels by extracellular epitope tagging in HeLa cells expressing GluA1_o_ alone (CTRL) or co-expressing GluA1_o_ and CNIH-2 (CNIH-2). Representative micrographs show an increase in extracellularly HA-tagged GluA1_o_ on the cell surface of HeLa cells when CNIH-2 is co-expressed, visualized by anti-HA immunocytochemistry in non-permeabilized cells. Histogram data are mean surface expression levels ± SEM normalized to CTRL. Asterisk marks a significant difference from CTRL (p<0.01, unpaired Student's t-test; n = 24 for CTRL and CNIH-2, respectively). **B** Surface biotinylation of HeLa cells expressing GluA1_o_ (CTRL) or co-expressing GluA1_o_ and CNIH-2 (CNIH-2) (n = 6). Note that CNIH-2 co-expression increases both total and surface AMPAR levels. T = total, S = surface, I = internal. Protein load for S is concentrated 10fold. Depletion of the ER-resident lectin calnexin in S serves as a control for specificity of surface membrane biotinylation. **C** (Top) Representative current traces from somatic outside-out patches evoked by 0.5 mM glutamate (+250 µM TCM to block receptor desensitization) in sham-infected control and CNIH-2 over-expressing CA1 pyramidal neurons of organotypic hippocampal slice cultures DIV 7–10. (Bottom) Quantification of steady-state currents. Data are mean ± SEM. Asterisk marks a significant difference from control (p<0.0001, unpaired Student's t-test; n = 14 and n = 11 for CTRL and CNIH-2, respectively).

Sparked by our initial observation that co-expression of CNIH-2 enlarges the surface population of GluAs [22], we sought to characterize this effect in more detail. First, the amount of surface GluA1_o_ protein was quantified in the presence and absence of CNIH-2 expression using both an extracellular epitope tagging approach and surface membrane biotinylation. In the first approach, a haemagglutinin epitope, inserted into the extracellular N-terminal domain of GluA1_o_, was immunostained in HeLa cells without membrane permeabilization. As shown in Figure 1A, co-expression of CNIH-2 increased the steady-state amount of GluA1_o_ protein on the cell surface by a factor of 13.6±1.0 (n = 24; p<0.01). This effect was specific for GluA, as surface expression of the non-interacting potassium channel Kir2.1 was not affected by co-expression of CNIH-2 (data not shown). For the second experimental approach, all surface membrane proteins of HeLa cells expressing GluA1_o_ in the presence or absence of CNIH-2 were biotinylated, affinity-purified by streptavidin-coated beads, and finally target proteins were detected and quantified by immunoblot analysis. Figure 1B shows a representative Western blot revealing a significant increase in GluA1_o_ surface protein upon co-expression of CNIH-2. Intriguingly, also the total amount of GluA1_o_ increased in CNIH-2 co-expressing cells.

Next we tested whether the CNIH-2-mediated increase in GluA surface protein observed in heterologous expression systems is also true for native AMPARs in neurons. CNIH-2 was over-expressed in CA1 pyramidal neurons of organotypic hippocampal slice cultures and functional AMPAR surface expression was evaluated by quantifying glutamate-evoked currents in somatic outside-out patches in the presence of the desensitization blocker trichlormethiazide. Compared to sham-infected control neurons (0.96±0.12 nA; n = 14), CNIH-2 over-expression doubled current amplitudes (2.12±0.25 nA; n = 11; p<0.0001) (Fig. 1C). These results demonstrate that CNIH-2 promotes functional surface expression of AMPARs in both heterologous cells and primary neurons most likely due to a gain in the amount of surface protein. In addition, AMPAR currents could increase by CNIH-2-mediated modulation of their biophysical properties, i. e. an increase in single channel conductance [28].

### Subcellular distribution of CNIH-2

**Figure 2:**
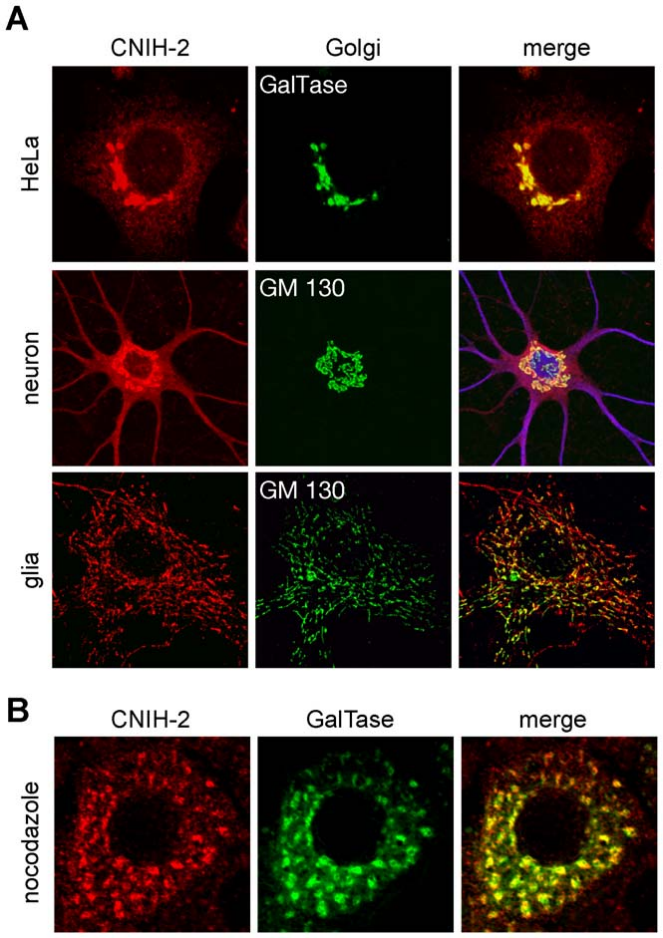
Subcellular localization of exogenously expressed CNIH-2. **A** Representative confocal images of HeLa cells, dissociated hippocampal neurons and glial cells over-expressing CNIH-2. Note the perinuclear accumulation of CNIH-2, which co-localizes with the Golgi markers GalTase-GFP and GM130. **B** CNIH-2 behaves similar to other Golgi-resident proteins cycling between Golgi and ER, as it co-distributes with GalTase into peripheral Golgi remnants upon nocodazole treatment (10 µM, 4 hrs).

To understand how CNIH-2 increased the surface population of AMPARs, we analyzed its subcellular distribution upon heterologous expression in HeLa cells and over-expression in dissociated hippocampal neurons and glial cells. Both in HeLa cells and hippocampal neurons (DIV 17), exogenously expressed CNIH-2 accumulated in a perinuclear compartment (Fig. 2A, upper and middle panel), while it exhibited a more punctate peripheral distribution in glial cells (Fig. 2A, lower panel). The compartment, in which CNIH-2 concentrated, could be identified as the Golgi complex by co-localization with the cis- or trans-Golgi marker proteins GM130 or galactosyltransferase (GalTase), respectively. In addition, incubation with the fungal toxin Brefeldin A (10 µg/ml, 30 min), which fuses Golgi membranes with those of the ER, resulted in a reversible redistribution of CNIH-2 into the ER (data not shown). With higher expression levels, we also observed CNIH-2 immunoreactivity that was homogenously distributed throughout the cells in a network-like pattern, most likely resembling the ER.

While so-called Golgi-resident proteins like galactosyltransferase show highest dwell times within the Golgi complex, they are in fact known to cycle within the early secretory pathway between ER and Golgi compartments [30], [31]. Microtubule depolymerization by nocodazole disrupts such cycling and induces the formation of multiple satellite Golgi stacks in close proximity to ER exit sites [30]. Figure 2B illustrates that in HeLa cells heterologously expressed CNIH-2 behaves like galactosyltransferase as it co-distributes into satellite Golgi stacks upon nocodazole treatment (10 µM, 4 hrs). Thus, exogenously expressed CNIH-2 localizes predominantly to the Golgi complex and behaves similar to other Golgi-resident proteins that cycle continuously between ER and Golgi complex.

### CNIH-2 promotes ER export of GluAs

**Figure 3:**
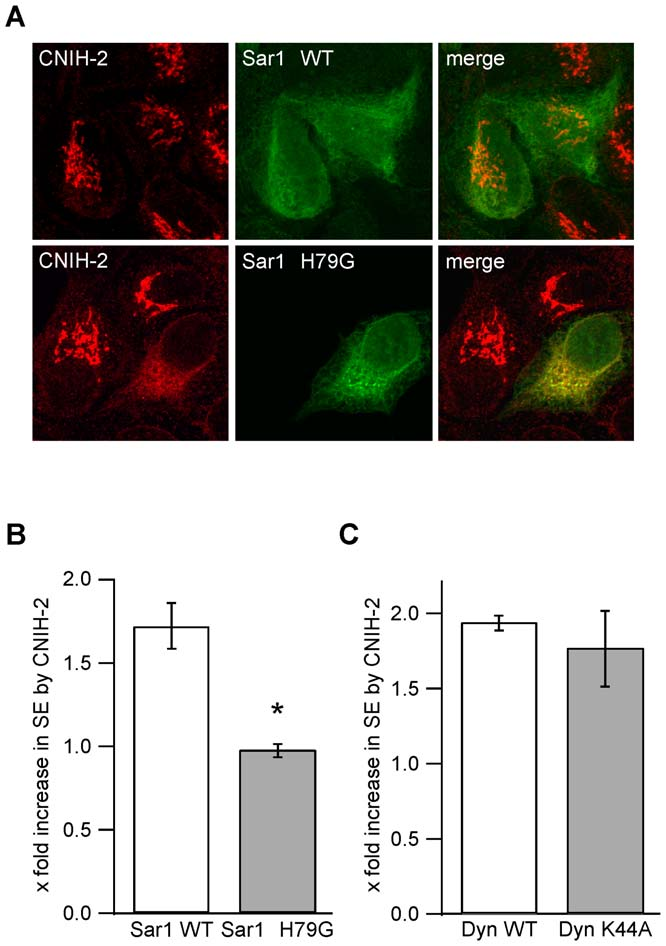
CNIH-2 facilitates ER export of AMPARs. **A** Representative confocal images of OK cells stably expressing CNIH-2. Co-expression of dominant-negative Sar1 H79G prevents ER export of CNIH-2 leading to its redistribution into the ER. **B** Quantification of GluA1_o_ surface expression levels by extracellular epitope tagging in the presence of CNIH-2 and either wildtype (WT) Sar1 (white bar) or mutant Sar1 H79G (grey bar). Data are mean increases in surface expression levels by CNIH-2 ± SEM normalized to GluA1_o_+Sar1 WT or GluA1_o_+Sar1 H79G without CNIH-2, respectively. Asterisk marks a significant increase in surface expression of GluA1_o_ by co-expression of CNIH-2 (p<0.001, unpaired Student's t-test; n = 12 for both experimental groups). **C** Quantification of GluA1_o_ surface expression levels in the presence of CNIH-2 and either wildtype dynamin-1 (white bar) or dominant-negative dynamin-1 K44A (grey bar) inhibiting clathrin-dependent endocytosis [38]. Data are mean increases in surface expression levels by CNIH-2 ± SEM normalized as in B (n = 6 for both experimental groups).

Cornichon and its orthologues have previously been described as cargo transporters, exporting soluble growth factors of the epidermal growth factor (EGF) family from the ER [25]–[27]. Based on our finding that exogenously expressed CNIH-2 cycles between ER and Golgi, we next addressed the question whether CNIH-2 might also serve as an ER cargo exporter for AMPA receptors. For this purpose, we used a heterologous expression system as the molecular mechanisms of selective ER export are conserved in all eukaryotic cells from yeast to mammalian cells including neurons [32]–[35]. Moreover, heterologous cells can be transfected at much higher rates than neurons allowing us to manipulate ER export of proteins and consecutively quantify their average surface expression in a representative number of cells. In opossum kidney (OK) cells stably expressing CNIH-2, ER export was blocked by transfection with a dominant-negative mutant of the small Ras-like GTPase Sar1 (Sar1 H79G) [36]. This constitutively active mutant of Sar1 prevents un-coating of transport vesicles, blocking ER export by inhibiting recycling of COPII components [37]. In OK cells expressing mutant Sar1 H79G, CNIH-2 was retained in the ER, while in neighboring non-transfected cells expressing endogenous wildtype Sar1, the accumulation of CNIH-2 in the Golgi remained unchanged (Fig. 3A). Thus, CNIH-2 is selectively exported from the ER in a COPII-dependent manner.

We then asked whether selective ER export of CNIH-2 is a prerequisite for increasing surface expression of GluAs. GluA1_o_ and CNIH-2 were co-expressed in HeLa cells with either wildtype Sar1 or the H79G mutant and surface expression was quantified using the extracellular epitope tagging approach. As shown in Figure 3B, CNIH-2 increased the surface expression of GluA1_o_ by a factor of 1.7±0.1 (n = 12; p<0.001) in the presence of wildtype Sar1, while this increase was effectively prevented in cells co-expressing Sar1 H79G (1.0±0.04; n = 12; p = 0.732). Surface expression of GluA1_o_ alone was not affected by co-expression of Sar1 H79G (1.01±0.07; n = 9, p = 0.234; data not shown). For reasons of cell toxicity brought about by the Sar1 mutant, data had to be acquired 16 hrs post transfection leading to significantly lower overall expression of GluA1_o_ than observed in previous experiments (Fig. 1). Within the time frame of 16 hrs, we did neither observe changes in cell morphology nor a reduction in GluA1 total protein expression. As the amount of surface protein is not only determined by the rate of anterograde transport, but also by the rate of removal from the plasma membrane, we probed a possible role for CNIH-2 in GluA endocytosis. Blocking of clathrin-dependent endocytosis via expression of a dominant-negative mutant of dynamin-1 (K44A; [38]) increased GluA1_o_ surface expression by a factor of 2.3 (n = 6; p<0.01) in the absence of CNIH-2 and by a factor of 2 (n = 6; p<0.001) in the presence of CNIH-2 (data not shown). However, the CNIH-2-mediated relative increase in GluA1_o_ surface expression was not affected by inhibition of endocytosis (Fig. 3C).

Taken together, these results clearly support the idea that CNIH-2 promotes the anterograde transport of GluAs by acting as an ER cargo exporter rather than a mere folding assistant in the ER.

### CNIH-2 affects glycosylation of surface GluA

GluAs are N-glycosylated at several consensus sites within their extracellular domains [39]. The degree of complexity of this posttranslational modification, as reflected by differential resistance against glycosidase digestion, is often used as an indicator of protein maturation during transport along the secretory pathway. Here, we analyzed the effect of CNIH-2 co-expression on the glycosylation pattern of GluA2 instead of GluA1, as the extent of N-glycosylation of GluA2 was more prominent in HeLa cells than the one of GluA1.

**Figure 4:**
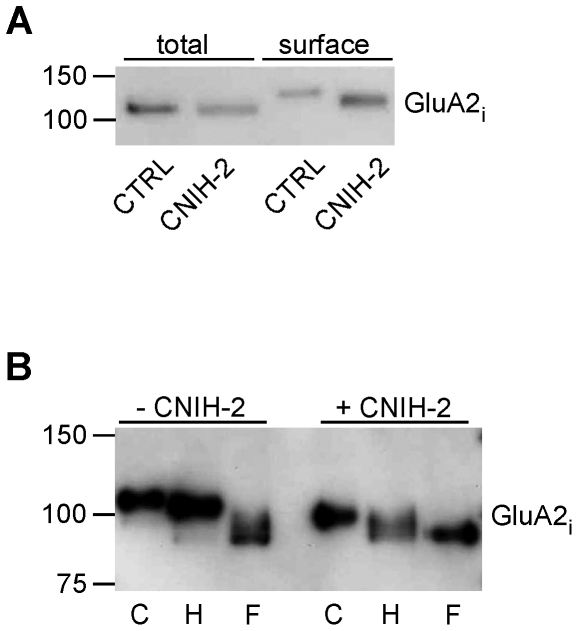
CNIH-2 changes glycosylation of GluAs. **A** Western blot analysis of total and surface populations of GluA2_i_ extracted from HeLa cells by surface biotinylation in the absence (CTRL) or presence of CNIH-2 (CNIH-2) (n = 4). Extensive glycosylation of surface GluA2_i_ during maturation increases its apparent molecular weight. Note the smaller increase upon co-expression of CNIH-2. **B** Enzymatic deglycosylation analysis of GluA2_i_ surface populations in the presence (+) or absence (−) of CNIH-2. Surface GluA2_i_ remained either untreated (C) or was incubated with either endoglycosidase H (H) or PNGase F (F). Note that upon CNIH-2 co-expression, the GluA2_i_ surface population remains sensitive to endoglycosidase H (n = 2).

As shown in Figure 4A, co-expression of CNIH-2 reduced the apparent molecular weight of surface GluA2_i_ isolated by surface biotinylation. To investigate whether the shift in mass was due to differences in glycosylation, we tested surface GluA2 receptors assembled in the absence or presence of CNIH-2 for their sensitivities to treatment with endoglycosidase H (Endo H) and PNGase F. While Endo H selectively cleaves high-mannose oligosaccharides, PNGase F removes all glycosylations [40]. As shown in Figure 4B, surface GluA2 receptors formed upon co-expression with CNIH-2 ran at smaller apparent MW and retained sensitivity to Endo H, while the surface population of homomeric GluA2 was only sensitive to treatment by PNGase F. This suggested that in HeLa cells CNIH-2 promotes export of GluA2 protein with an immature glycosylation pattern.

### CNIH-2-mediated trafficking of GluAs is isoform-specific

**Figure 5:**
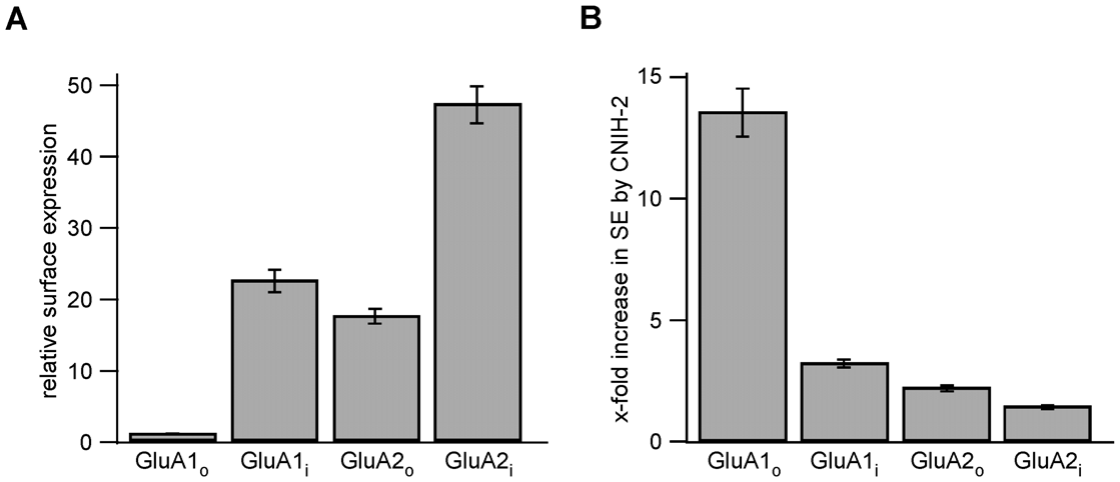
Surface trafficking of GluAs by CNIH-2 is splicing-dependent. **A** Quantification of GluA surface expression levels by extracellular epitope tagging in HeLa cells expressing the indicated GluA subunits. Data are mean ± SEM normalized to GluA1_o_ (GluA1_o_: n = 24; GluA1_i_: n = 9; GluA2_o_: n = 12; GluA2_i_: n = 12). **B** Increase in GluA surface expression mediated by CNIH-2 in HeLa cells. Data are mean ± SEM normalized to surface expression of respective GluA subunits without CNIH-2 (GluA1_o_: n = 24; GluA1_i_: n = 8; GluA2_o_: n = 12; GluA2_i_: n = 12).

Several studies have demonstrated that ER export of GluAs critically depends on isoform and flip/flop splice variant [9], [41], [42]. We therefore addressed the question whether the increase in GluA surface population by CNIH-2 was similarly affected by using the extracellular epitope tagging approach. As shown in Figure 5A, the ability of GluA1 and GluA2 subunits to reach the cell surface of HeLa cells varied markedly with GluA2_i_ being the subunit that was expressed most and GluA1_o_ the one being expressed least on the cell surface in the absence of CNIH-2. In the presence of CNIH-2, the increase in surface expression of indicated GluA subunits was exactly opposite (Fig. 5B). It was largest for the flop variant of GluA1 (GluA1_o_; 13.6±1.0; n = 24) and smallest for the flip isoform of GluA2 (GluA2_i_; 1.4±0.1; n = 12). GluA1_i_ and GluA2_o_ showed intermediate increases in surface expression when co-expressed with CNIH-2 (3.2±0.2 (n = 8) and 2.2±0.1 (n = 12), respectively). These data demonstrate that CNIH-2 can at least partially compensate for splice form-dependent differences in the ER export rates of GluA isoforms.

### Interaction with AMPARs converts CNIH-2 into a surface membrane protein

CNIH-2 has been shown to considerably modify the gating properties of heterologously expressed AMPARs - an observation rather unexpected for a cycling cargo exporter as it implies association of CNIH-2 with GluAs at the plasma membrane [16], [22], [28]. We, therefore, investigated surface localization of CNIH-2 in both heterologous cells and hippocampal neurons.

**Figure 6:**
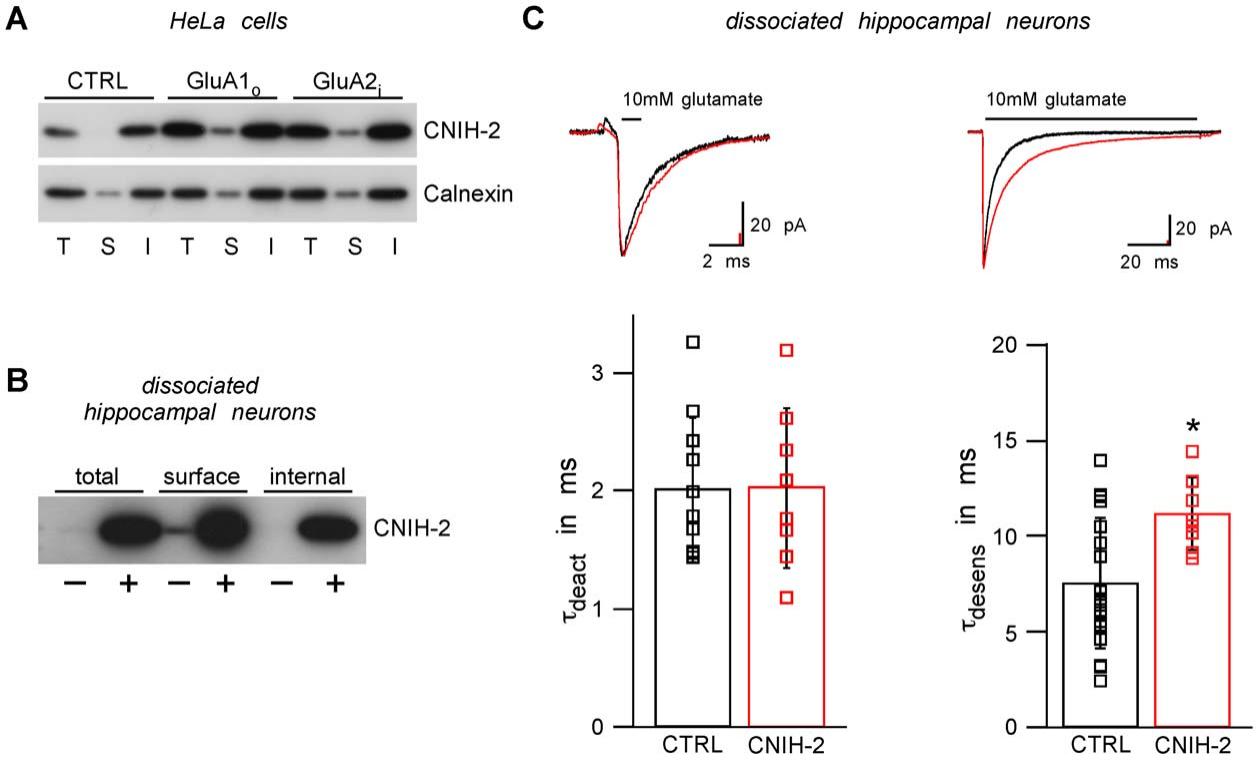
CNIH-2 is rendered a surface membrane protein by assembly with AMPARs. **A** Total (T), surface (S) and internal (I) populations of CNIH-2 in HeLa cells expressing either CNIH-2 alone (CTRL) or together with GluA1_o_ or GluA2_i_, respectively. S is concentrated 10fold. Note that in the absence of GluAs, CNIH-2 cannot be detected in the surface fraction. However, it is robustly observed in the plasma membrane when co-expressed with GluAs (n = 4). **B** Total (T), surface (S) and internal (I) populations of CNIH-2 in dissociated hippocampal neurons (DIV 17) transduced with CNIH-2 (+) or GFP (−). S is concentrated 10fold. Both endogenous (−) and over-expressed (+) CNIH-2 can be detected on the cell surface (n = 5). **C** (Top) Representative current traces recorded in somatic outside-out patches excised from dissociated hippocampal neurons (DIV 16–21) over-expressing either GFP (CTRL, black) or CNIH-2 (CNIH-2, red) upon 1 ms (left panel) and 100 ms applications (right panel) of 10 mM glutamate. (Bottom) Quantification of deactivation and desensitization kinetics. Data are given as mean ± SD. Asterisk denotes a significant difference from control (p<0.01, unpaired Student's t-test; deactivation: n = 10 and 8 for CTRL and CNIH-2, respectively; desensitization: n = 19 and 8 for CTRL and CNIH-2, respectively).

Surface biotinylation revealed that heterologously expressed CNIH-2 could only be found on the cell surface when GluA subunits were co-expressed (Fig. 6A). In the absence of GluA subunits, even high expression levels of CNIH-2 were not sufficient to drive detectable amounts of CNIH-2 to the plasma membrane. Thus, only upon interaction with AMPARs, CNIH-2 leaves the ER-to-Golgi cycle and is rendered a surface membrane protein. In line with this result, both endogenous and exogenously over-expressed CNIH-2 was detected on the cell surface of dissociated hippocampal neurons, which express endogenous GluAs (Fig. 6B).

The observation of CNIH-2 protein on the cell surface of primary neurons was further corroborated by functional recordings using somatic outside-out patches from dissociated hippocampal neurons that were virally transduced with CNIH-2. As shown in Fig. 6C, the currents elicited by fast application of 10 mM glutamate displayed a significantly slower time course of desensitization (τ_desens_ = 11.1±1.9 ms; n = 8; p<0.01) than those obtained from sham-infected neurons (τ_desens_ = 7.5±3.4 ms; n = 19). Deactivation kinetics were not affected by over-expression of CNIH-2 (Fig. 6C).

Taken together, these data indicate that the interaction with AMPARs has converted CNIH-2 from a sole cargo exporter promoting anterograde trafficking into a surface membrane protein modifying the signaling of the receptors.

## Discussion

In the present study, we have investigated the role of the mammalian cornichon homologue CNIH-2 in AMPAR processing in both heterologous as well as primary cells. We show that CNIH-2 interacts with GluAs early in the secretory pathway and promotes COPII-dependent ER export of the receptors. As a consequence, CNIH-2 increases the density of functional AMPARs on the cell surface of heterologous cell lines and neurons. Moreover, our study demonstrates for the first time that mammalian CNIH-2 escapes from its evolutionarily conserved subcellular localization behavior that is cycling between the ER and Golgi complex and reaches the cell surface when accompanied by GluA subunits. Thus, AMPA receptors commandeer the cargo exporter CNIH-2 for use as a bona fide auxiliary subunit, which is then able to modify both AMPAR trafficking and gating.

### The cargo exporter CNIH-2

The prototype of the cornichon protein family CNIH-1 and its orthologues throughout the eukaryotic phylogenetic tree have been described as ER cargo exporters for soluble growth factors of the TGFα family and the integral membrane protein Axl2p [25]–[27], [43], [44]. Our initial observation that co-expression of the mammalian cornichon homologue CNIH-2 robustly increased the functional AMPAR surface density suggested a functional role very similar to the one of CNIH-1 and prompted us to characterize its subcellular distribution in more detail.

Upon exogenous expression in HeLa cells and over-expression in hippocampal neurons and glial cells, we find CNIH-2 to accumulate in the early secretory pathway with a preferential localization to the trans-Golgi complex. Just like other Golgi-resident proteins, however, CNIH-2 is not literally Golgi-resident. It cycles between the trans-Golgi and the ER compartments as demonstrated by co-segregation with β-1,4-galactosyltransferase into satellite Golgi stacks, when ER-to-Golgi transport was disturbed [30], [31]. The predominant probability of CNIH-2 localization to the Golgi complex depends on its selective export from the ER initiated by COPII coat protein complex formation [45], as interference with COPII function by co-expression of a dominant-negative Sar1 mutant redistributed CNIH-2 into the ER. Simultaneously, the increase in surface expression of co-expressed GluA receptors was abolished showing that COPII-dependent trafficking of CNIH-2 is also a prerequisite for its function in cargo transport. Thus, our results are fully consistent with a role for mammalian CNIH-2 as an ER cargo exporter: it could take up cargo proteins within the ER, mediate their preferential export by interacting with the COPII coat, then might release their cargo in the Golgi complex and finally cycle back to the ER to take up new cargo. Additional evidence for the hypothesis that mammalian CNIH-2 increases the surface density of GluA receptors by facilitating their ER export is provided by the observation that CNIH-2 co-expression did not only increase the amount of GluA on the cell surface but also in total cell lysates. Protein homeostasis in the ER can be modeled as a balance between three interacting pathways: ER-assisted protein folding, export of proteins from the ER and their ER-associated degradation [46]. Thus, if ER export of GluA is enhanced by CNIH-2, its ER-associated degradation will be less engaged explaining the reported increase in total amount of GluA protein. Finally, we could exclude the retrograde transport of GluA subunits being affected by CNIH-2, as dominant-negative inhibition of clathrin-dependent endocytosis of GluA did not preclude the increase in surface expression by CNIH-2 co-expression.

### The cargo exporter becomes an auxiliary AMPAR subunit

Using surface biotinylation assays in HeLa cells, we demonstrate that CNIH-2 can escape from the cycle within the early secretory pathway between ER and Golgi compartments. If assembled with GluA subunits, CNIH-2 is transported to the cell surface. In line with these observations from heterologous expression systems, both endogenous and over-expressed CNIH-2 is readily detected in the plasma membrane of dissociated hippocampal neurons that express native AMPARs (Fig. 6). Moreover, electrophysiological recordings indicate that over-expressed CNIH-2 is able to modify AMPAR gating in these neurons. Thus, we propose that the interaction with GluA subunits let CNIH-2 evolve from an ER cargo exporter into a bona fide auxiliary subunit of AMPARs. Unlike other cargo, GluA subunits are not disengaged from CNIH-2 during early anterograde traffic, but stay together for surface transport. This might be a novel property in the evolutionary diversification of the mammalian cornichon family of proteins. In this respect, it is noteworthy that CNIH-1, which has the highest sequence homology to the cornichon orthologues in yeast and drosophila, was not found as a constituent of native AMPARs [22].

CNIH-1 has been assigned not simply a facilitating but a regulatory role on ER export of TGF-α, crucially depending on its expression level [27]. Immature TGF-α was prevented from anterograde traffic by high expression levels of CNIH-1. The authors explained their finding by preferential interaction of CNIH-1 with the less glycosylated forms of TGF-α, while implying a definite localization of CNIH-1 in the early secretory pathway. Intriguingly, we also found less complex glycosylation of GluA2_i_ when co-expressed with CNIH-2. However, those immature receptors were not retained in the ER as might be expected, but reached the plasma membrane efficiently. We did also not observe any negative effect on GluA surface expression when increasing the cDNA transfection ratio of CNIH-2:GluA2_i_ (data not shown). Hence, we interpret our results on GluA maturation by sterical hindrance that is imposed on putative N-glycosylation sites in the glutamate receptor by its interaction with the extracellular loop of CNIH-2 [16]. Whether this is of physiological relevance for AMPAR stability on the cell surface or for their biophysical properties, e.g. their ligand affinity [47], needs to be investigated.

### Conclusion

In summary, our cell biological experiments demonstrate that the mammalian cornichon homologue CNIH-2 has an evolutionarily preserved function as a cargo exporter promoting COPII-dependent export from the ER. Interaction with AMPARs, however, significantly extends the physiological role of CNIH-2: while still exploiting CNIH-2 as a cargo exporter for adjustment of imbalances in splice form-dependent trafficking, AMPARs wrest CNIH-2 from its cycle between ER and Golgi complex and integrate them into their functional complexes on the cell surface. Thus, they commandeer CNIH-2 for use as a bona fide auxiliary subunit.

## Materials and Methods

### Molecular biology

Genebank accession numbers of cDNAs used are: NM_001025132 (CNIH-2), M38060.1 (GluA1_i_), NM_031608.1 (GluA1_o_), NM_017261.2 (GluA2_i_), NP_001077280.1 (GluA2_o_). All cDNAs were verified by sequencing. Dynamin-1 K44A [38] was a gift from S. L. Schmid, β-1,4-galactosyltransferase fused to GFP-A206K (GalTase-GFP; [30]) was generously provided by J. Lippincott-Schwartz.

### Cell culture

HeLa cells (DSMZ) were grown in DMEM (Invitrogen) supplemented with 10% fetal calf serum (Biochrom), 1% HEPES (Invitrogen) and 1% penicillin/streptomycin (Invitrogen) at 37°C and 5% CO_2_. At ∼80% confluence, cells were transfected with the respective cDNAs using Fugene HD Transfection Reagent (Roche, Promega) following the supplier's directions. Opossum kidney cells (American Type Culture Collection) were grown in DMEM-F12 (Invitrogen) supplemented with 10% fetal calf serum (Biochrom) and 1% penicillin/streptomycin (Invitrogen). At ∼80% confluence, cells were transfected with the respective cDNAs using Lipofectamine 2000 (Invitrogen) following the supplier's directions. Brefeldin A (BFA; Sigma) and nocodazole (Sigma) were applied at 10 µg/ml and 10 µM, respectively.

Primary cultures of hippocampal neurons were obtained from rats at embryonic age E 18. The entire hippocampus was isolated and dissociated with trypsin. Cells were plated in a 24-well plate at a density of 50.000 cells/well on poly-D-lysine coated coverslips. Cells were grown in glia-conditioned Neurobasal medium supplemented with 2% B27, 1% Na-pyruvate, 1% glutamax, 1% penicillin/streptomycin, 1% Fungizone (all Invitrogen) at 37°C and 5% CO_2_. At days 12–14 in vitro (DIV 12–14), cells were transduced with CNIH-2 by high-titer lentiviral preparations or at DIV 15–20 by semliki forest viral particles.

Standard procedures were used to prepare organotypic hippocampal slice cultures from rats at postnatal age P 7–9 [48]. In brief, animals were decapitated, hippocampi were rapidly isolated, and transversally chopped in 400 µm thick slices using a McIllwain tissue chopper. Isolation was done in dissection medium containing 100 ml MEM (EBSS, 25 mM HEPES) (Invitrogen), 1 ml penicillin/streptomycin, 1 ml 1 M Tris buffer, pH 7.2. After 30 min recovery at 4°C slices were placed onto Millicell cell culture inserts (Millipore). Medium contained 100 ml MEM, 1 ml 200 mM L-glutamine, 50 ml HBSS and 50 ml horse serum.

### Immunocytochemistry

Cells were fixed in 4% paraformaldehyde in phosphate-buffered saline (PBS) for 10 min at 4°C and pre-treated with 10% normal goat serum (NGS, Calbiochem) in PBS with 0.04% Triton X-100 (PBS-T) for 1 hour at room temperature (RT) to prevent unspecific antibody-binding. Then cells were incubated with the respective primary antibodies in 2% NGS in PBS-T for 1 hour at RT (rabbit anti-CNIH-2, 1∶250, [26]; mouse anti-GM130, 1∶100, BD Transduction Laboratories; chicken anti-MAP2, 1∶10.000, Abcam). Immunoreactivity was finally visualized by secondary anti-mouse, anti-rabbit and anti-chicken antibodies conjugated to cy-2, cy-3, or cy-5 (1∶250 in 10% NGS in PBS-T, Dianova).

### Imaging

Cells were imaged with a confocal laser scanning microscope (LSM510, Zeiss) using the following excitation wavelengths and filter settings. EGFP, cy-2: Ar-laser (488 nm), BP505–530 nm; cy-3: HeNe-laser (543 nm), LP560 nm; cy-5: HeNe-laser (633 nm), BP690–750 nm.

### Quantification of surface expression of proteins

Extracellular epitope tagging was performed as described previously [22]. All steps were performed at RT and in the absence of detergents. Briefly, transfected HeLa cells grown to confluency in 34 mm dishes were fixed in 4% paraformaldehyde in PBS for 20 min, pre-treated with 10% NGS in PBS for 1 hour and incubated with a primary mouse anti-HA-antibody (1∶100, Santa Cruz) followed by goat anti-mouse secondary antibody conjugated to horseradish peroxidase (1∶5000 in 10% NGS in PBS, Santa Cruz). Immunoreactivity was detected by enzymatic turnover of SuperSignal ELISA Femto Maximum Sensitivity Substrate (Thermo Scientific) and quantified in a Glomax 20/20 n luminometry system (Promega). Test and control dishes were always processed in parallel to correct for differences in staining efficiency between experiments. Data are given as mean ± SEM, expressed as relative surface expression levels of the respective control. Statistically significant differences were assessed using the unpaired Student's t-test. For surface biotinylation, living confluent HeLa cells or living dissociated hippocampal neurons were washed three times with ice-cold PBS and biotinylated for 10 min on ice using membrane impermeable EZ-link Sulfo-NHS-SS-biotin (HeLa: 0.1 mg/ml in PBS, neurons: 0.15 mg/ml in PBS; Thermo Scientific). To quench the remaining unbound biotin, cells were washed two times with ice-cold PBS supplemented with 50 mM NH_4_Cl and once with ice-cold PBS alone. Cells were harvested, lysed by sonification, and crude membrane fractions were isolated by ultracentrifugation at 125,000× g for 20 min. Membrane protein complexes were solubilized in ComplexioLyte buffer 91 (LOGOPHARM GmbH) for 30 min at 4°C followed by ultracentrifugation (15 min at 125,000× g). Solubilisates were incubated with streptavidin agarose resin (Thermo Scientific) for 1 hour at 4°C to separate biotinylated proteins. After washing with PBS, biotinylated proteins were eluted by incubation in Laemmli buffer for 10 min at 37°C. Protein samples were finally resolved by SDS-PAGE and identified by Western blotting.

### Deglycosylation assay

Solubilized membrane or surface protein fractions were denatured in 50 mM phosphate buffer, supplemented with 1% SDS and 0.5 M β-mercaptoethanol and afterwards incubated with endoglycosidase H or PNGase F (both Roche) in the presence of 0.5% NP-40 and protease inhibitors overnight at 37°C. Protein samples were finally resolved by SDS-PAGE and Western blotting.

### SDS-PAGE and Western Blotting

Protein samples were run on 12% SDS-PAGE. After electroblotting on PVDF membrane (Millipore), Western analysis was performed using rabbit anti-CNIH-2 (1∶1000; [26]), mouse anti-calnexin (1∶1000, Abcam), rabbit anti-GluA1 (1∶1000, Millipore), mouse anti-GluA2 (1∶1000, Millipore) followed by goat anti-mouse or anti-rabbit secondary antibodies conjugated to horseradish peroxidase (1∶15000, Santa Cruz). Blots were finally developed with ECL plus (GE Healthcare).

### Preparation of viral particles

CNIH-2:IRES:GFP was cloned into pSCA1 [49]. Semliki forest viral particles were produced by standard procedures [48]. CNIH-2 was cloned into the HIV-derived lentiviral vector CMV-GFP [50]. High-tighter lentivirus preparations were produced by standard procedures [51]. Used virus solutions had a titer between 10^7^ and 10^9^.

### Electrophysiology

Recordings from organotypic hippocampal slice cultures: Slices were used between DIV 7 and DIV 10. Somatic outside-out patches from CA1 pyramidal cells were performed at RT and clamped at −70 mV using a Multiclamp 700B amplifier. Signals were low-pass filtered at 2.5 kHz and sampled at 10 kHz with a DigidData 1322. AMPAR currents were evoked by local application of 0.5 mM glutamate for 2 s in the presence of 250 µM trichlormethiazide to block receptor desensitization. Recordings were made within 24 hours after infection, using 2–3 MΩ glass electrodes filled with an internal solution consisting of the following (in mM): 115 CsMeSO_3_, 20 CsCl, 10 HEPES, 2.5 MgCl_2_, 4 Na_2_-ATP, 0.4 Na-GTP, 10 Na-phosphocreatine, 0.6 EGTA, and 0.1 spermine, pH 7.2. External perfusion medium consisted of (in mM): 119 NaCl, 2.5 KCl, 2.5 CaCl_2_, 1.3 MgSO_4_, 2.7 MgCl_2_, 1 NaH_2_PO_4_, 26.2 NaHCO_3_ and 11 glucose, saturated with 95% O_2_ and 5% CO_2_. Data were analyzed with Clampfit 10.0 and Prism 5.0.

Recordings from dissociated hippocampal neurons: Electrophysiological recordings from outside-out patches excised from cultured hippocampal neurons (DIV 16–21) were performed at RT and a holding potential of −120 mV. Recordings were made within 24 hours after infection with an Axopatch 200B amplifier, low-pass filtered at 10 kHz, and sampled at 50–100 kHz. Pipettes made from quartzglass had resistances of 1–2 MΩ when filled with intracellular solution (in mM): 135 CsF, 33 CsOH, 2 MgCl_2_, 1 CaCl_2_ and 11 EGTA, pH 7.4. Extracellular solution applied to outside-out patches was composed as follows (in mM): 5.8 KCl, 144 NaCl, 0.9 MgCl_2_, 1.3 CaCl_2_, 0.7 NaH_2_PO_4_, 5.6 D-Glucose, and 10 HEPES, pH 7.4. Rapid application/removal of glutamate (10 mM dissolved in extracellular solution) was performed with a piezo-controlled fast application system with a double-barrel application pipette that enables solution exchanges within less than 100 µs (20–80%, measured by switching the open tip of the patch pipettes between normal and 10fold-diluted extracellular solution). Deactivation and desensitization of AMPARs were characterized by time constants derived from bi-exponential fits to the decay phase of the respective currents; weighted tau (τ_w_) was calculated as τ_w_ = (τ_f_ * a_f_)+(τ_s_ * a_s_), where a_f_ and a_s_ are the relative amplitudes of the fast (τ_f_) and slow (τ_s_) exponential components. Quality of the fit result was judged from the χ^2^ deviations. Curve fitting and further data analysis were done with Igor Pro 4.05A Carbon.
